# Predicting the structure and vibrational frequencies of ethylene using harmonic and anharmonic approaches at the Kohn–Sham complete basis set limit

**DOI:** 10.1007/s00894-015-2902-z

**Published:** 2016-01-22

**Authors:** Aneta Buczek, Teobald Kupka, Małgorzata A. Broda, Adriana Żyła

**Affiliations:** Faculty of Chemistry, University of Opole, 48, Oleska Street, 45-052 Opole, Poland; Department of Biosystematics, University of Opole, Oleska 22, 45-052 Opole, Poland; Institute of Physics, Adam Mickiewicz University in Poznan, 65, Umultowska Street, 61-614 Poznań, Poland

**Keywords:** Ethylene, Anharmonic vibration, VPT2, DFT, CBS, Polarization-consistent basis sets, Correlation-consistent basis sets

## Abstract

In this work, regular convergence patterns of the structural, harmonic, and VPT2-calculated anharmonic vibrational parameters of ethylene towards the Kohn–Sham complete basis set (KS CBS) limit are demonstrated for the first time. The performance of the VPT2 scheme implemented using density functional theory (DFT-BLYP and DFT-B3LYP) in combination with two Pople basis sets (6-311++G** and 6-311++G(3df,2pd)), the polarization-consistent basis sets pc-*n*, aug-pc-*n*, and pcseg-*n* (*n* = 0, 1, 2, 3, 4), and the correlation-consistent basis sets cc-pV*X*Z and aug-cc-pV*X*Z (*X* = D, T, Q, 5, 6) was tested.

The BLYP-calculated harmonic frequencies were found to be markedly closer than the B3LYP-calculated harmonic frequencies to the experimentally derived values, while the calculated anharmonic frequencies consistently underestimated the observed wavenumbers. The different basis set families gave very similar estimated values for the CBS parameters. The anharmonic frequencies calculated with B3LYP/aug-pc-3 were consistently significantly higher than those obtained with the pc-3 basis set; applying the aug-pcseg-*n* basis set family alleviated this problem. Utilization of B3LYP/aug-pcseg-*n* basis sets instead of B3LYP/aug-cc-pV*X*Z, which is computationally less expensive, is suggested for medium-sized molecules. Harmonic BLYP/pc-2 calculations produced fairly accurate ethylene frequencies.

**Electronic supplementary material:**

The online version of this article (doi:10.1007/s00894-015-2902-z) contains supplementary material, which is available to authorized users.

**Graphical Abstract:**
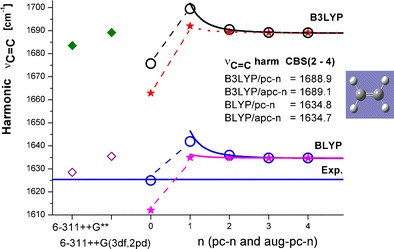
In this study, the performance of the VPT2 scheme implemented using density functional theory (DFT-BLYP and DFT-B3LYP) in combination with the polarization-consistent basis sets pc-*n*, aug-pc-*n*, and pcseg-*n* (*n* = 0, 1, 2, 3, 4), and the correlation-consistent basis sets cc-pVXZ and aug-cc-pVXZ (*X* = D, T, Q, 5, and 6) was tested. For the first time, we demonstrated regular convergence patterns of the structural, harmonic, and VPT2-calculated anharmonic vibrational parameters of ethylene towards the Kohn–Sham complete basis set (KS CBS) limit

## Introduction

Theoretical modeling of structures and vibrational frequencies has often been performed in combination with analysis of experimental IR and Raman spectra. Ethylene is the smallest compound with a carbon–carbon double bond, and can be considered a simplified model of polyenes [1, 2]. The local environments of the C=C bonds present in numerous natural and synthetic compounds, including polyenes in red corals, have routinely been studied using vibrational spectroscopy [2].

Vibrational frequencies calculated using SCF, DFT, and MP2 are overestimated because the effects of anharmonicity are omitted [3–5]. For example, SCF predicts C–H, N–H, and O–H stretching vibrations that are about 5–10 % higher than those actually observed. This deficiency in SCF, DFT, and MP2 has been pragmatically corrected by introducing frequency scaling [4, 6, 7]. Unfortunately, the method of calculation and the basis set are important considerations during the tedious estimation of proper, empirically derived scaling factors.

Instead of using scaling factors, vibrational analysis based on the inclusion of an anharmonic correction is better justified theoretically. Among several anharmonic approaches (including VSCF [8–11], VCI [12–14], and VCC [15]), the recent implementation of VPT2 [16–20] has proven to be very robust and efficient when investigating small and medium-sized molecules. Obviously, such an anharmonic approach is significantly more computationally expensive than a simple harmonic model. For this reason, anharmonic calculations are mainly performed using DFT [21, 22] and relatively small basis sets, such as Pople basis sets or early members of the series of Dunning's correlation-consistent basis sets [23–26].

A family of correlation-consistent basis sets, cc-pVXZ, and another similar family with additional augmented diffuse functions (aug-cc-pVXZ), have been proposed by Dunning [23–26]. The main feature of these basis sets is the regular and smooth convergence of atomic and molecular energies towards the complete basis set (CBS) limit. Moreover, it has been shown that the CBS values for energy and some other parameters that are directly related to the energy can be estimated using two- and three-parameter formulae. The best agreement between these theoretically calculated values and the corresponding experimentally derived values was obtained when the results calculated using the most complete basis sets (*X* > 2) were fitted. Two additional basis set series that are similar to those of Dunning are available: the polarization-consistent [27–32] and XZP [33] basis sets. The same limiting parameter values (CBS) were reportedly [34–36] obtained from noncorrelated and correlated calculations performed using Dunning’s and Jensen’s basis sets. It is important to note that the results converge more quickly when polarization-consistent basis sets are applied.

Correlation-consistent basis sets have been used to accurately calculate the geometries and vibrational parameters of several small molecules at the coupled cluster level of theory [37, 38]. Approaches based on correlation-consistent basis sets and coupled cluster calculations are very expensive computationally and are thus unsuitable for larger molecular systems. Unfortunately, the systematic calculation of molecular systems using polarization-consistent basis sets is not yet commonplace. Besides, the combination of anharmonic models for frequency calculations with polarization-consistent basis sets is still the source of debate.

Another issue that needs to be explored is the convergence pattern toward the CBS limit of Kohn–Sham wavelengths calculated using the pc-*n* and cc-pVXZ basis sets. In our recent studies [39–42], we demonstrated that H_2_O, CH_2_O, and H_2_NCHO harmonic and anharmonic frequencies converged regularly toward the KS limit when Jensen-type basis sets were used. These basis sets were originally designed as general-purpose basis sets to be used in particular to predict Hartree–Fock and Kohn–Sham energies and structural and vibrational parameters. In subsequent studies [42], we noticed that anharmonic frequencies showed irregular behavior in polar solvents when the PCM solvation model was used [43]. In addition, we observed [41] a simple relation between harmonic and anharmonic C=O stretching frequencies (the corresponding wavenumbers differ by about 31 cm^−1^).

Although a number of studies on the structure and harmonic properties of ethylene have been reported [38, 44–47], there has been no systematic use of polarization-consistent basis sets, particularly the newly designed segmented contracted ones, at the DFT level of theory. Thus, due to the importance of ethylene as the basic building block of linear polyenes, we thought that it would be interesting to find an efficient way of accurately calculating its structural and vibrational properties. Therefore, in the study reported in the present paper, we compared the accuracy of the harmonic and anharmonic frequencies of ethylene in the gas phase calculated using recently developed families of Jensen basis sets with the results obtained using traditional Dunning basis sets. We tested the performance of the harmonic model, and the computationally expensive anharmonic approach, using a well-performing hybrid density functional (B3LYP). The performance of B3LYP in predicting ethylene frequencies was compared to the results obtained with the BLYP functional [48], benchmark literature values, and reported experimental data.

## Computational methods

All DFT calculations using the popular B3LYP [49–51] and BLYP [50–52] density functionals in combination with 6-311++G** and 6-311++G(3df,2pd) Pople-type basis sets [53, 54], Jensen’s polarization-consistent pc-*n*, aug-pc-*n* [28, 29], pcseg-*n*, and aug-pcseg-*n* [55] (for* n* = 0, 1, 2, 3, 4) basis sets, and Dunning’s correlation-consistent cc-pVXZ and aug-cc-pVXZ [23–26] basis sets were performed with the D.01 version of the Gaussian 09 program [56]. The Jensen basis sets were downloaded from EMSL [57–59]. It is important to note that all of the B3LYP-calculated and BLYP-calculated structural parameters and harmonic frequencies of ethylene obtained using the A.02 [60] version of Gaussian 09 were practically identical to those obtained using the D.01 [56] version. However, the anharmonic frequencies for ethylene obtained with the latter version were somewhat smaller [48], resulting in better agreement of the B3LYP(anharmonic)-predicted C=C stretching frequency with the corresponding experimental value.

Unconstrained geometry optimization of ethylene was performed using very tight convergence criteria and a very large grid, as specified using the keyword INT(GRID = 150590). Harmonic and anharmonic frequencies were then calculated. The anharmonic wavenumbers were obtained via Barone’s implementation of vibrational second-order perturbation theory (VPT2) [18] in the latest (D.01 [56]) version of Gaussian 09. Detailed assignment of ethylene vibrational modes was performed based on the results of previous studies [61–63] (see also Table S1 in the “Electronic supplementary material,” ESM).

Moreover, to assess the performance of the chosen density functional in predicting structural and vibrational parameters of ethylene, calculations using the pc-*n*, aug-pc-*n*, cc-pVXZ, and aug-cc-pVXZ basis sets were performed, and the individual results, *Y*(*x*), were extrapolated toward the Kohn–Sham (KS) complete basis set limit *Y*(∞) using the three-parameter [64, 65] and two-parameter [66, 67] formulae

1 $$ Y(x)=Y\left(\infty \right)+A \exp \left(-x/B\right) $$

2 $$ Y(x)=Y\left(\infty \right)+A/{x}^3. $$

The extrapolated value *Y*(∞) is the optimal estimate of the predicted structural or vibrational parameter [34, 39, 40, 67, 68] for a very large (e.g., infinite) zeta or cardinal number *x*, and *A* and *B* are fitting parameters. In addition, to check the convergence of the anharmonic ν(C=C) mode for ethylene, the calculations were repeated using the newly introduced segmented contracted polarization-consistent basis sets pcseg-*n* and aug-pcseg-*n* [55]. A similar strategy has been used in previous studies [34, 39, 69, 70] to accurately predict the structural, vibrational, and NMR parameters of small molecules at the HF, DFT, MP2, and CCSD(T) levels of theory at the complete basis set limit (CBS).

Please note that, for the sake of brevity, the basis sets cc-pVXZ, aug-cc-pVXZ, pc-*n*, aug-pc-*n*, pcseg-*n*, and aug-pcseg-*n* are sometimes abbreviated to “XZ,” “aXZ,” “pcn,” “apcn,” “pcsegn,” and “apcsegn,” respectively, in the figures for this paper.

## Results and discussion

### Structure of ethylene

The structure of ethylene was fully optimized using the B3LYB and BLYP density functionals in combination with the two fairly complete and flexible Pople-type basis sets 6-311++G** and 6-311++G(3df,2pd), a selected series of Jensen’s polarization-consistent basis sets, and Dunning's correlation-consistent basis sets. Next, using Eq. 1 or 2, the CBS values of the structural parameters of ethylene were estimated from the last two (or three) points obtained for the corresponding largest Jensen and Dunning basis sets. The resulting structural parameter values are compared in Table 1 with the best corresponding values given in the literature [44–46], including those for the semiempirical structure [47] of C_2_H_4_.

**Table 1:** Structural parameters of ethylene, calculated using the BLYP and B3LYP density functionals in combination with selected basis sets

Method/basis set	C=C length (Å)	C–H length (Å)	C–C–H angle (°)
B3LYP			
6-311++G**	1.3289	1.0850	121.74
6-311++G(3df,2pd)	1.3247	1.0823	121.74
CBS (pc-*n*)	1.3240	1.0815	121.73
CBS (aug-pc-*n*)	1.3239	1.0815	121.74
CBS (pcseg-*n*)	1.3241	1.0818	121.73
CBS (aug-pcseg-*n*)	1.3241	1.0818	121.74
CBS (cc-pVXZ)	1.3241	1.0818	121.74
CBS (aug-cc-pVXZ)	1.3241	1.0818	121.74
BLYP			
6-311++G**	1.3384	1.0915	121.79
6-311++G(3df,2pd)	1.3341	1.0887	121.79
CBS (pc-*n*)	1.3335	1.0877	121.78
CBS (aug-pc-*n*)	1.3334	1.0877	121.79
CBS (cc-pVXZ)	1.3337	1.0882	121.79
CBS (aug-cc-pVXZ)	1.3337	1.0882	121.79
From the literature:			
Best composite theory^a^	1.3307	1.0809	121.44
Best composite theory^b^	1.3308	1.0803	121.40
** Semi-empirical** ^**c**^	**1.3305(10)**	**1.0805(10)**	**121.45(10)**

The CBS values were estimated using a two-parameter fit (Eq. 2)

^a^ From [44];^ b^ from [46];^ c^ from [47];

**Fig. 1a–c:**
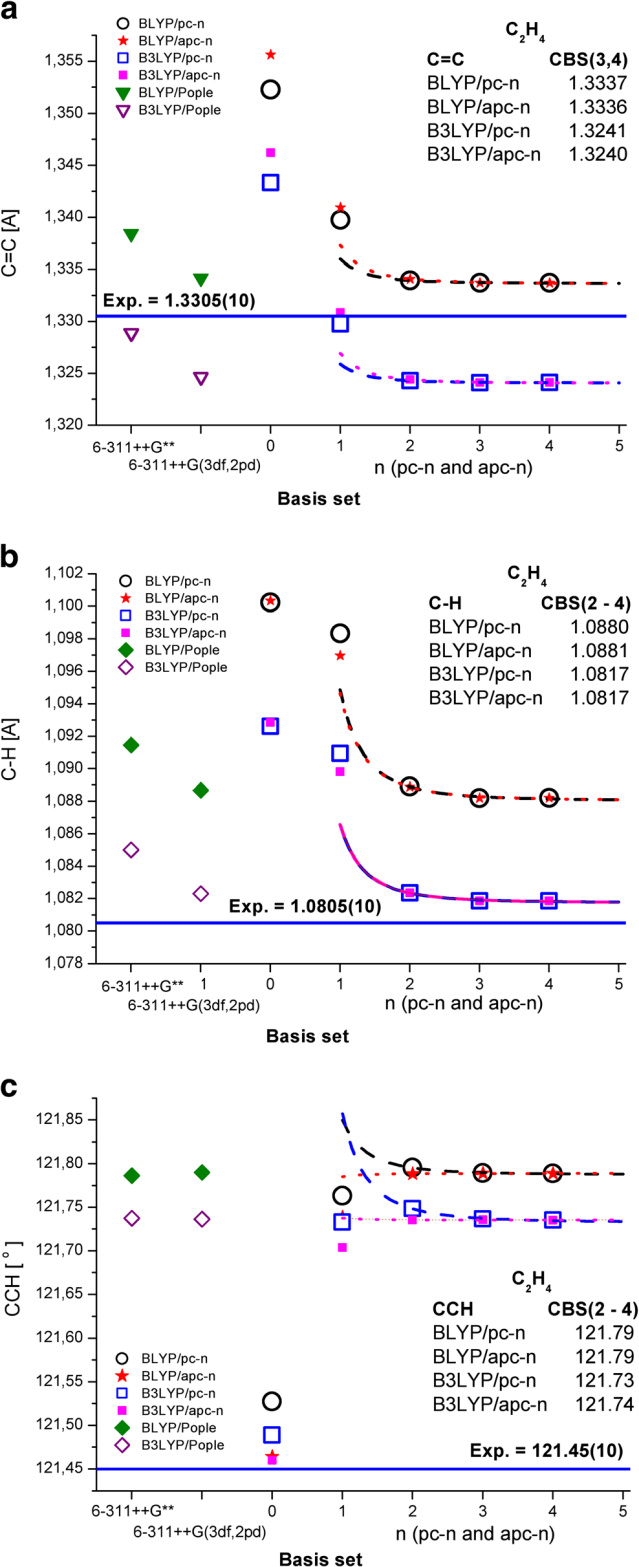
Sensitivity of the BLYP- and B3LYP-calculated ethylene C=C (**a**) and C–H (**b**) bond lengths, as well as the C–C–H bond angle (**c**), to the quality of the basis set.* Continuous lines* mark experimental values [47], and the results of CBS fits are also shown

Figure 1 shows the C–C and C–H bond lengths and C–C–H angle of ethylene obtained with the 6-311++G**, 6-311++G(3df,2pd), pc-*n*, and aug-pc-*n* basis sets. The best fits, as well as the estimated CBS values, obtained using Eq. 2 with the three largest basis sets are also shown. The C–C bond length was found to be insensitive to the basis set size, while the C–H bond and C–C–H angle did not fully converge when pc-2 and aug-pc-2 were used. All of the calculated structural parameters are gathered in Table S2A, B in the ESM.

It is clear from Fig. 1 that the structural parameters of ethylene calculated using the BLYP and B3LYP density functionals almost converged when the pc-2 and aug-pc-2 basis sets were applied, but the results were far from the estimated CBS values (i.e., accidentally over- or underestimated) when smaller basis sets were used. Augmenting the basis set with diffuse functions did not improve the results (e.g., compare the pc-3 data with the aug-pc-3 data, or the pc-4 data with the aug-pc-4 data).

As expected, at the complete basis set limit, the values of all of the structural parameters depended only on the density functional used (the pc-*n*, aug-pc-*n*, pcseg-*n*, and aug-pcseg-*n* series of basis sets all gave very similar results; see Table 1 and Table S2A, B in the ESM for the results obtained for all of the basis sets considered in this work). For example, the differences between estimates given by B3LYP and BLYP at the Kohn–Sham limit in combination with different families of basis sets for the lengths of the C–C and C–H bonds in ethylene are 0.0002, 0.0003, and 0.0003, 0.0005 Å, while the estimates afforded by these two functionals for the C–C–H angle differ by only 0.01°.

The B3LYP functional underestimates the C=C bond length (given that the reference semiempirical value [47] is 1.3305 Å) by 0.007 Å, while BLYP overestimates the reference value by 0.003 Å. The corresponding deviations obtained when the smallest basis set is used (6-311++G**) are −0.002 and 0.008 Å, respectively. For the C–H bond length, the deviations of the B3LYP- and BLYP-calculated values from the reference value are 0.001 and 0.007 Å. Somewhat larger deviations are seen when the smallest basis set (6-311++G**) is applied: 0.005 and 0.011 Å. Interestingly, the hybrid B3LYP and pure BLYP density functionals yield very similar deviations regardless of the basis set used: they overestimate the C–C–H angle by 0.29° and 0.34°, respectively. Therefore, we can conclude that the results for the structural parameters of ethylene gathered in Table 1 indicate that B3LYP is somewhat better than BLYP at predicting the geometry of ethylene. It is important to note that the values of the structural parameters of ethylene almost converged when the pc-2 and aug-pc-2 basis sets were used. The 6-311++G** basis set, which can feasibly be applied to large molecules such as polyenes [1, 71], is somewhat less accurate than (computationally very demanding) CBS calculations.

### Convergence patterns of harmonic and anharmonic vibrations of ethylene

Due to differences in the anharmonicities of the normal modes of ethylene, high- and low-frequency harmonic vibrations are calculated with different levels of accuracy [45]. To permit a comparison of theoretically predicted frequencies with the corresponding observed frequencies, Table S1 in the ESM shows experimental (fundamental) frequencies for ethylene, their symmetries, and their assignments [62, 63, 72]. Among the 12 vibrations shown, the four high-frequency ones (due to C–H stretching) are overestimated to the greatest degree in comparison to the experimental values [62, 72]. The characteristic C=C stretch vibration is overestimated to a lesser degree, and the remaining modes (below 1500 cm^−1^) are expected to be predicted more accurately by theory.

To show the nonuniform accuracy of the vibrational frequency values predicted using the density functionals and basis sets studied in this work for both the harmonic and anharmonic (VPT2) models, we now consider individual vibrational frequency deviations and RMS deviations. For brevity, all of the frequency values are listed in Tables S3A–S8A in the ESM. Moreover, all of the calculated vibrational frequencies were plotted as a function of basis set size, and the corresponding CBS values were estimated using the two largest basis sets (these are not shown here for the sake of brevity).

**Fig. 2a–b:**
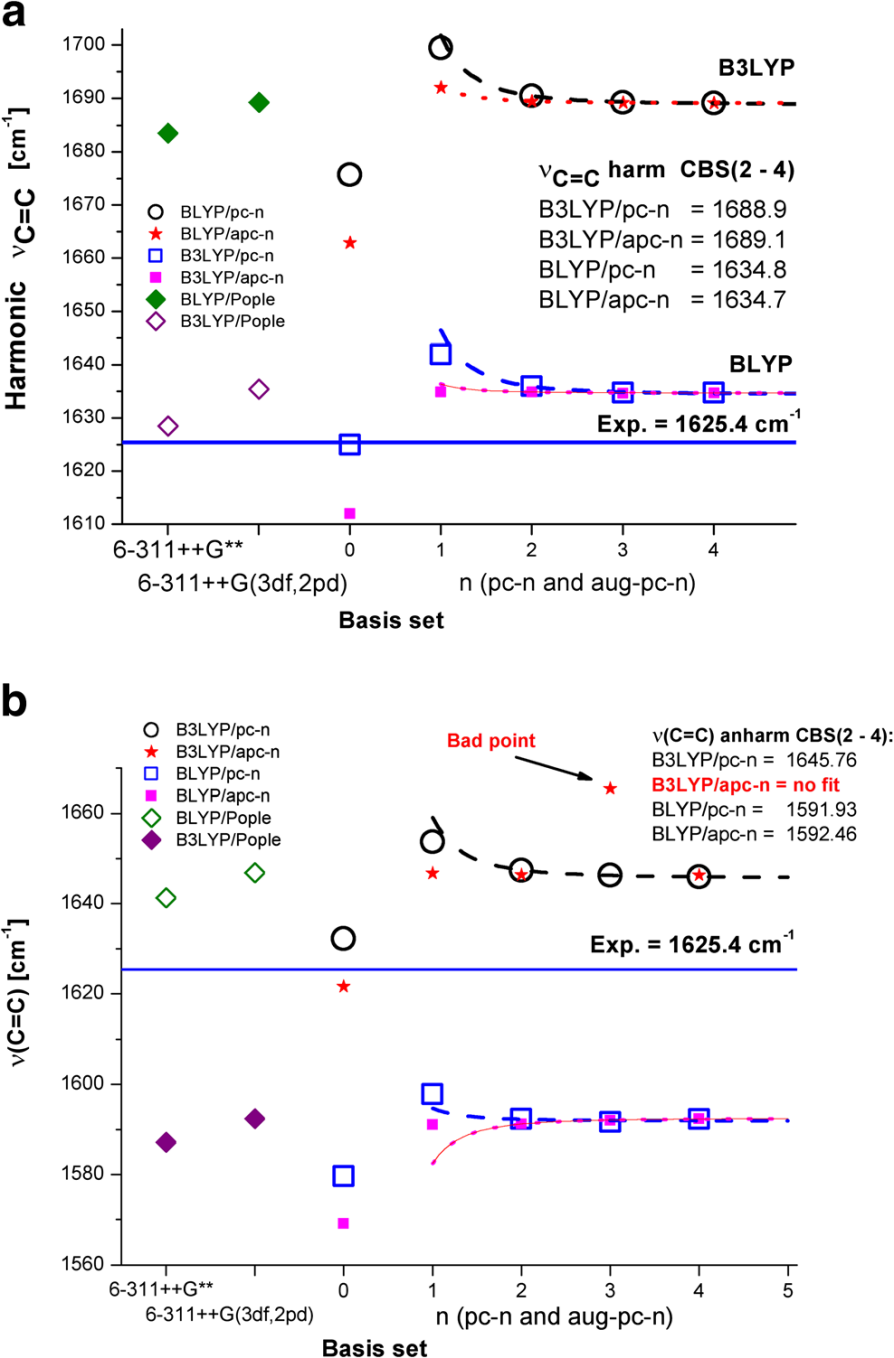
Sensitivity of the BLYP- and B3LYP-calculated values for the ethylene ν(C=C) vibrational frequency to the quality of the basis set when predictions are made using the harmonic (**a**) and anharmonic (**b**) approximations. A* continuous line* indicates the experimental value [47], and the CBS fitting results are also shown

Since C=C stretching is the most important diagnostic mode in the vibrational (Raman) spectra of polyenes, we analyzed the performance of the selected theoretical models used to predict this mode. Figure 2a shows the influence of the basis set on the magnitude of C=C stretching; results were calculated using the B3LYP and BLYP density functionals in combination with the pc-*n* and aug-pc-*n* basis sets. In this case, the pc-*n* and aug-pc-*n* basis sets perform equally well for* n* ≥ 2. Results showing a considerable amount of scatter are obtained for* n * = 0 and 1, as well as for the two selected Pople-type basis sets. Table 2 presents the deviations in the harmonic and anharmonic CBS values of the C=C stretching vibrational frequency from the corresponding experimentally derived value (see also Tables S1 and S8B in the ESM). Note that the CBS values for the C=C stretching vibrational frequency are overestimated (by about 64 cm^−1^) by a simple harmonic model when using the B3LYP density functional (Fig. 2a and Table 2). On the other hand, employing the BLYP density functional with the pc-*n*, aug-pc-*n*, cc-pVXZ, and aug-cc-pVXZ basis sets leads to fairly accurate predictions for the harmonic C=C stretching vibrational frequency (the experimental value of 1625.4 cm^−1^ is overestimated by less than 10 cm^−1^).

**Table 2:** Deviations of calculated CBS values for the ethylene C=C stretching vibrational frequency from the corresponding experimentally derived value [62, 63, 72]

Basis set	BLYP		B3LYP	
	Harmonic	Anharmonic	Harmonic	Anharmonic
pc-*n*	9.36	−32.67	63.65	20.25
aug-pc-*n*	9.41	−32.80	63.70	7.00
cc-pVXZ	8.74	−37.26	63.74	23.27
aug-cc-pVXZ	9.14	−37.75	63.69	9.22
pcseg-*n*	-	-	63.58	18.31
aug-pcseg-*n*	-	-	63.63	16.94

By contrast, the BLYP-calculated anharmonic frequencies obtained using the pc-*n* and aug-pc-*n* basis sets (see Fig. 2b) are significant underestimates (about 33 cm^−1^ too low) compared to the experimentally derived value [62, 72]. On the other hand, the corresponding anharmonic B3LYP/CBS values obtained with the pc-*n* and aug-pc-*n* basis sets overestimate the actual (experimental) value by only about 20 and 7 cm^−1^, respectively. However, closer inspection of the latter results suggests that the B3LYP/aug-pc-3 result is about 20 cm^−1^ higher than the pc-3 result. Indeed, all 12 anharmonic frequencies of ethylene obtained at the B3LYP/aug-pc-3 level of theory are significantly overestimated in comparison with those obtained with the pc-3 basis set. Thus, two-point fitting cannot be performed in this case (this entry is marked in red in Fig. 2b). On the other hand, it is important to stress that the anharmonic frequencies calculated at the BLYP/aug-pc-*n* level of theory show the correct behavior (regular convergence).

This poor performance of B3LYP/aug-pc-3 when it was used to calculate anharmonic frequencies (see also Table S5B in the ESM) prompted us to check the performance of the newly released (and refined) pcseg-*n* and aug-pcseg-*n* basis sets [55], as well as Dunning’s cc-pVXZ and aug-cc-pVXZ series of basis sets [23–26]. The relevant results are gathered in Table S6A, B in the ESM (the ESM presents frequencies calculated with both of the density functionals considered in this work and all of the selected basis sets; see Tables S3A–S8A). None of the other basis sets exhibited the poor performance shown by B3LYP/aug-pc-3 when calculating anharmonic frequencies.

We are aware that the good performance of BLYP in predicting the ethylene ν(C=C) harmonic frequency could be an effect of fortuitous error cancelation [48]. Nevertheless, this density functional may represent a pragmatic choice for investigating a set of compounds containing C=C bonds. Thus, we consider the BLYP density functional to be suitable for fairly accurate modeling of the structures and harmonic vibrational parameters of larger polyenes. The abovementioned hypothesis is also supported by the results of our recent studies on difluorinated ethylene isomers [48] and perfluorinated compounds [73], which are used as precursors for plastic optical fibers (POF).

It is important to note that the accuracies of the methods and basis sets considered in Table 2 when they were used to predict the C=C stretching vibrational frequency are also valid for all other individual vibrations. Indeed, as shown in Table 3, when all vibrations are considered, the calculated RMS deviation is about 65 cm^−1^ when the BLYP anharmonic model is used, but only about 26 cm^−1^ when the BLYP harmonic model is employed. The reverse situation is observed for the B3LYP calculated harmonic and anharmonic frequencies (RMS devations of about 77 and 20 cm^−1^).

**Table 3:** RMS deviations of calculated CBS frequencies for ethylene from the corresponding experimentally derived frequencies [62, 63, 72]

Basis set	BLYP		B3LYP	
	Harmonic	Anharmonic	Harmonic	Anharmonic
pc-*n*	26.04	62.96	76.90	16.42
aug-pc-*n*	26.04	63.53	76.92	58.25^a^
cc-pVXZ	25.99	62.56	76.90	18.15
aug-cc-pVXZ	26.13	67.15	76.96	~31.5^a^
pcseg-*n*	-	**-**	76.86	20.87
aug-pcseg-*n*	-	**-**	76.83	23.63

^a^ The RMS deviations are high in these cases because the values obtained using aug-pc-3 are much too high or because there is considerable scatter in the results afforded by aug-cc-pVXZ

The current work considered only one molecular system (ethylene) and two density functionals (B3LYP and BLYP). Such a limited study cannot warrant extrapolating the results obtained here to other molecules. However, in the future it would be interesting to test our approach on a set of polyenes containing conjugated sets of double and single carbon–carbon bonds.

### Convergence patterns of the total and ZPV energies of ethylene

**Fig. 3:**
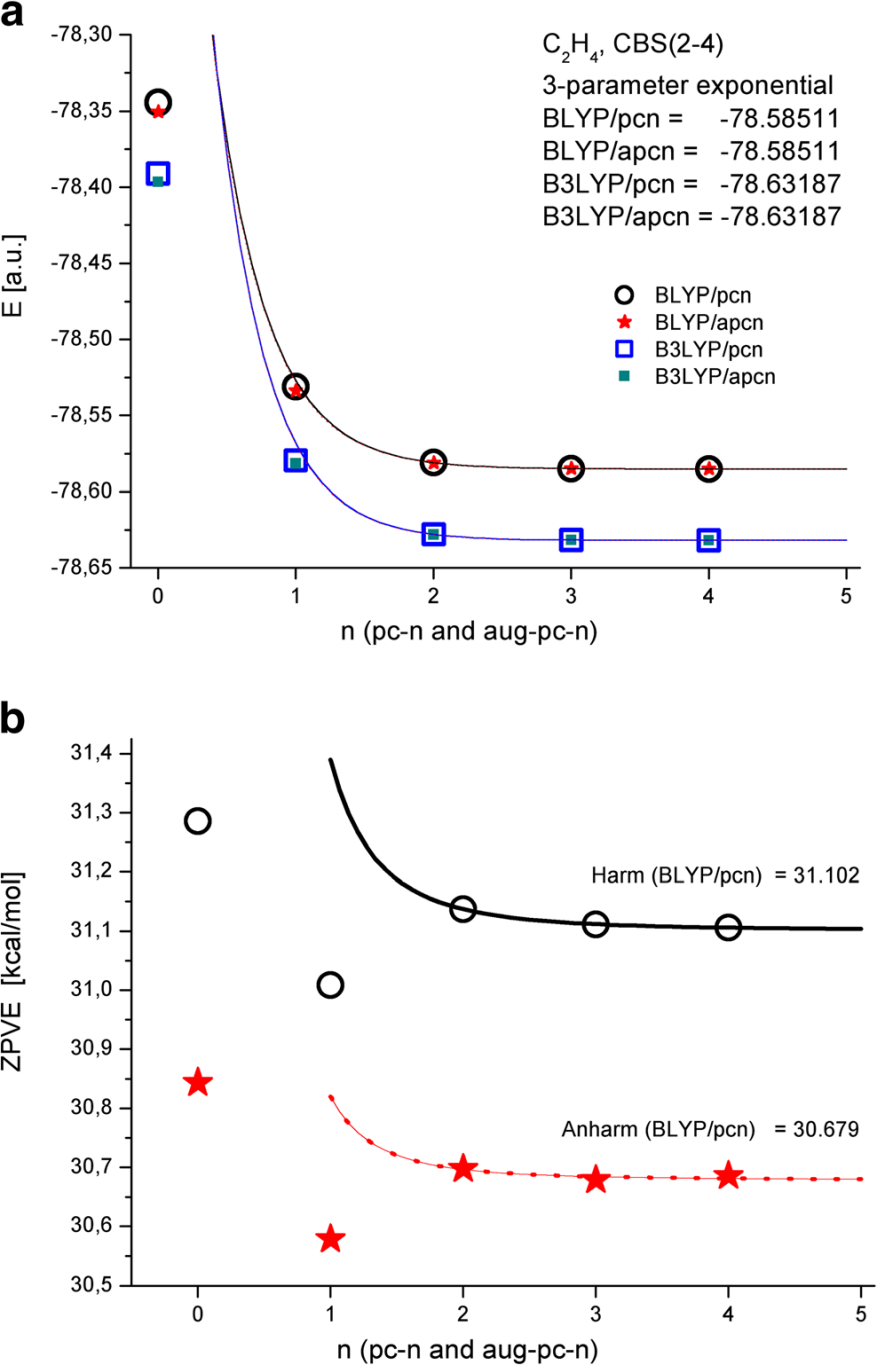
**a** Convergence of the BLYP- and B3LYP-calculated energies of ethylene toward the Kohn–Sham limit when using the pc-*n* and aug-pc-*n* basis set families. **b** Convergence of the raw and anharmonic ZPVE values calculated using BLYP/pc-*n*

The molecular energy and the zero-point vibrational energy are two of the most commonly encountered parameters in theoretical thermochemistry [5, 23, 26, 67, 74]. Obviously, since we have considered different calculation methods (for example, density functionals) in this work, we are not interested in absolute energy values, only energy differences. The correlation-consistent basis sets developed by Dunning and coworkers were designed to show the regular convergence of molecular energy and parameters directly related to energy toward the complete basis set limit in correlated calculations [23–26, 67, 68]. Among the CBS-estimated parameters are structural, magnetic, and vibrational parameters. Since these parameters are calculated as derivatives (or second derivatives) of the energy, the convergence curves are not very smooth, so the results for the initial (smallest) basis sets are often not used in the CBS fitting procedure [26, 34, 70, 75]. Thus, we expected the convergence of the ZPV energy of ethylene to be significantly worse than the convergence of its total energy. Figure 3a shows the influence of the basis set on the B3LYP- and BLYP-calculated energies of ethylene. In this case, a three-parameter exponential fit based on the results obtained using the three largest basis sets (*n* = 2, 3, 4) was employed. Note that the least accurate result, obtained using the* n* = 0 basis set, is clearly far from the fit line. Also note that the CBS energies calculated with the pc-*n* and aug-pc-*n* basis sets were identical.

The corresponding BLYP-calculated raw and anharmonic ZPV energies show similar patterns starting from* n* = 2 (Fig. 3b). The three-parameter fit lines based on the results obtained using the three largest basis sets are also plotted. The CBS values of the ZPV energy obtained using a simply harmonic model are overestimated (by about 1.3 %). A similar relation between the raw and anharmonic ZPV values is observed when the B3LYP density functional is employed (not shown). Table 4 lists the estimated CBS values of the raw and anharmonic ZPV energies, calculated using the BLYP and B3LYP density functionals and the selected basis set families. The DFT values are compared with benchmark CCSD(T)/aug-cc-pVQZ results reported in the literature [76]. As also seen for the absolute energy of ethylene, the ZPV values calculated with B3LYP and BLYP differ. The raw ZPV energies calculated with the B3LYP density functional and different basis sets are larger than the corresponding anharmonic values. The same pattern is observed for the BLYP-derived ZPV energies.

**Table 4:** Calculated CBS values of the raw and anharmonic ZPV energies (kcal/mol)

Basis set	BLYP		B3LYP	
	Harmonic	Anharmonic	Harmonic	Anharmonic
cc-pVXZ	31.112	30.678	31.957	31.567
aug-cc-pVXZ	31.109	30.587	31.567	~31.5^a^
pc-*n*	31.361	30.941	32.208	31.776
aug-pc-*n*	31.368	30.957	32.216	31.796^a^
pcseg-*n*	-	-	31.937	31.518
aug-pcseg-*n*	-	-	31.944	31.528
From the literature:				
CCSD(T)/aQZ	32.048^b^			

^a^ There was scatter in some of the B3LYP/aug-cc-pVXZ results, and the aug-pc-4 result is shown for aug-pc-*n* because the aug-pc-3 result is significantly larger than the pc-3 result

^b^ The CCSD(T)/aQZ-calculated harmonic result from NIST is shown for comparison [76]

### CPU times required for harmonic and anharmonic calculations

The frequency analysis presented above shows that the CBS-calculated RMS deviations in the vibrational frequencies of ethylene are nearly the same for all of the basis set families. In the last stage of our study, we probed the computational demands of each model (harmonic and anharmonic), density functional, and basis set (Table 5). It is apparent from Table 5 that employing Dunning basis sets (particularly the augmented versions) instead of the polarization-consistent ones is significantly more computationally expensive.

**Table 5:** CPU times (in days, hours, and minutes) required for ethylene optimization and anharmonic frequency calculations performed using the B3LYP density functional along with Dunning and segmented polarization-consistent basis sets

		Number of basis functions		CPU time^a^			
				Optimization		Anharmonic frequencies	
Dunning basis sets	*X*	cc-pV*X*Z	aug-cc-pV*X*Z	cc-pV*X*Z	aug-cc-pV*X*Z	cc-pV*X*Z	aug-cc-pV*X*Z
	2	48	82	8 m	8 m	1 h 47 m	3 h 35 m
	3	116	184	14 m	16 m	6 h 3 m	15 h 44 m
	4	230	344	29 m	54 m	1 d 2 h 17 m	3 d 13 h 26 m
	5	402	574	1 h 48 m	6 h 59 m	6 d 5 h 20 m	30 d 19 h 37 m
	6	644	886	13 h 51 m	4 d 15 h 34 m	53 d 1 h 37 m	226 d 19 h 14 m
		Number of basis functions		Optimization		Anharmonic frequencies	
Polarization-consistent basis sets	*n*	pcseg-*n*	aug-pcseg-*n*	pcseg-*n*	aug-pcseg-*n*	pcseg-*n*	aug-pcseg-*n*
	0	26	38	7 m	6 m	1 h 7 m	1 h 27 m
	1	48	82	7 m	9 m	1 h 44 m	3 h 28 m
	2	116	184	15 m	19 m	5 h 54 m	15 h 49 m
	3	252	366	34 m	1 h 2 m	1 d 6 g 3 h	4 d 2 h 6 m
	4	446	618	2 h 41 m	1 d 27 m	8 d 4 h 27 m	42 d 15 h 45 m

^a^ Calculations were performed using 24 processors and 50 GB of memory

## Conclusions

Structural parameters of ethylene were calculated using the BLYP and B3LYP density functionals. The estimated complete basis set (CBS) values were found to be the same regardless of the polarization-consistent or correlation-consistent basis set used. The C=C and C–H bond lengths and the C–C–H angle practically converged when basis sets with* n* = 2 and* X* = 3 were used, and augmentation with diffuse functions did not change the CBS values. The B3LYP functional was somewhat better at predicting the structure of ethylene.

The RMS deviations of 12 CBS-estimated vibrational frequencies of ethylene from their experimentally derived values depended on the density functional used and whether a correction for anharmonicity was included. We demonstrated that the BLYP-calculated harmonic frequencies had RMS deviations that were about 3 times smaller than those of the B3LYP-calculated harmonic frequencies. Using the computationally expensive VPT2 model significantly improved the performance of B3LYP (the original RMS deviation of 77 dropped to 20 cm^−1^), but led to BLYP-calculated results that underestimated the actual value even more than before (original RMS deviation of 26 increased to 65 cm^−1^).

When the B3LYP density functional was used, the pc-4 basis set yielded near-identical anharmonic frequencies to those given by the aug-pc-4 basis set; the same was true for the basis sets with* n* = 2, too. However, the results provided by aug-pc-3 were systematically overestimated (by about 20 cm^−1^ in the case of the C=C stretching vibrational frequency) with respect to the results given by pc-3. This deficiency suggests that CBS frequencies cannot be reliably estimated using B3LYP/aug-pc-*n* calculations. There was some scatter in the anharmonic frequencies and the ZPV energy values calculated using the B3LYP/cc-pVXZ and B3LYP/aug-cc-pVXZ methods, making CBS frequency estimation unreliable. On the other hand, the newly refined aug-pcseg-*n* series of basis sets produced regularly converging anharmonic frequencies. The Dunning basis sets yielded the same results, but required much more CPU time.

A future study aimed at checking the performance of the segmented polarization-consistent basis set family and the B3LYP density functional when using the VPT2 model to accurately reproduce the experimental vibrational spectra for a set of short polyenes of general formula H–(C=C–C=C)_*n*_–H (* n* = 1, 2, 3, 4) is needed. In addition, the use of harmonic BLYP/pc-2 calculations as a computationally less expensive alternative to VPT2 calculated at the B3LYP/pc-2 level of theory should be tested as a means of predicting trends in C=C stretching frequencies for polyenes with* n* values of up to 8.

## Electronic supplementary material

ESM 1 The online version of this article contains supplementary material, which is available to authorized users. (DOC 385 kb)
