# Supplementary material for: Predicting the structure and vibrational frequencies of ethylene using harmonic and anharmonic approaches at the Kohn–Sham complete basis set limit
Source: J Mol Model. 2016 Jan 22;22:42. doi: 10.1007/s00894-015-2902-z (PMC4723629; doi:10.1007/s00894-015-2902-z)
Supplement: Supplementary file 1 — The online version of this article contains supplementary material, which is available to authorized users. (DOC 385 kb) [file 894_2015_2902_MOESM1_ESM.doc]

**Supplementary material for:**

Aneta Buczek*^a^, Teobald Kupka*^a^, Małgorzata A. Broda^a^, and Adriana Żyła^abc^

^a^Faculty of Chemistry, University of Opole, 48, Oleska Street, 45-052 Opole, Poland;

^b^Department of Biosystematics, University of Opole, Oleska 22, 45-052 Opole, Poland; cPresent address: Adam Mickiewicz University in Poznan, Institute of Physics, 65, Umultowska Street, 61-614 Poznań, Poland

**Tab. S1.** Observed fundamental frequencies of ethylene^a^ with indicated mode, symmetry and assignment.

| Fundamental | Mode | Assignment |
| --- | --- | --- |
| 3104.89 | 9 (B_2u_) | CH_2_ stretch |
| 3083.36 | 5 (B_3g_) | CH_2_ stretch |
| 3022.03 | 1 (A_g_) | CH_2_ stretch |
| 2988.64 | 11 (B_1u_) | CH_2_ stretch |
| 1625.4 | 2 (A_g_) | CC stretch |
| 1442.47 | 12 (B_1u_) | CH_2_ scissor |
| 1343.54 | 3 (A_g_) | CH_2_ scissor |
| 1222 | 6 (B_3g_) | CH_2_ rock |
| 1025.59 | 4 (A_u_) | CH_2_ twist |
| 948.77 | 7 (B_3u_) | CH_2_ wag |
| 939.86 | 8 (B_2g_) | CH_2_ wag |
| 825.93 | 10 (B_2u_) | CH_2_ rock |

a) From ref. 62, 63, 72

**Tab. S2A.** Structural parameters (in Å and deg) and harmonic and anharmonic stretch C=C mode of ethylene (in cm^-1^), calculated with BLYP and B3LYP density functionals and several basis sets. The deviations from experimental values and the number of basis functions and CBS values for the largest Jensen basis sets (estimated from Eq. 1 and 2) are also given.

| BASIS SET | | **BLYP** | | | | | **B3LYP** | | | | | | |
| --- | --- | --- | --- | --- | --- | --- | --- | --- | --- | --- | --- | --- | --- |
|  |  | Structure | | | str C=C | | Structure | | | | str C=C | |  |
|  |  | C=C | C-H | HCH | harm | anharm | C=C [Å] | C-H [Å] | HCH | | harm | anharm | b.f. |
| **6-311++G**** | | 1.3384 | 1.0915 | 121.79 | 1628.54 | 1587.18 | 1.3289 | 1.0850 | 121.74 | | 1683.54 | 1641.27 | 72 |
| 6-311++G (3df,2pd) | | 1.3341 | 1.0887 | 121.80 | 1635.46 | 1591.71 | 1.3247 | 1.0823 | 121.74 | | 1689.25 | 1646.41 | 138 |
| pc-0 | | 1.3523 | 1.1002 | 121.53 | 1624.99 | 1579.62 | 1.3434 | 1.0926 | 121.49 | | 1675.72 | 1632.24 | 26 |
| pc-1 | | 1.3400 | 1.0984 | 121.76 | 1641.93 | 1597.78 | 1.3300 | 1.0910 | 121.73 | | 1699.48 | 1653.73 | 48 |
| pc-2 | | 1.3340 | 1.0889 | 121.80 | 1635.99 | 1592.35 | 1.3243 | 1.0824 | 121.75 | | 1690.50 | 1647.42 | 116 |
| pc-3 | | 1.3337 | 1.0882 | 121.79 | 1634.77 | 1591.65 | 1.3241 | 1.0819 | 121.74 | | 1689.24 | 1646.31 | 264 |
| pc-4 | | 1.3337 | 1.0882 | 121.79 | 1634.78 | 1592.28 | 1.3241 | 1.0819 | 121.74 | | 1689.13 | 1645.93 | 470 |
| **CBS(pcn)** | | **1.3335** | **1.0877** | **121.784** | **1633.84** | **1591.89** | **1.3240** | **1.0815** | **121.73** | | **1688.16** | **1645.99** |  |
| aug-pc-0 | | 1.3556 | 1.1004 | 121.46 | 1612.07 | 1569.0884 | 1.3462 | 1.0929 | 121.46 | | 1662.91 | 1621.64 | 38 |
| aug-pc-1 | | 1.3410 | 1.0970 | 121.74 | 1634.96 | 1591.10 | 1.3309 | 1.0898 | 121.70 | | 1692.03 | 1646.74 | 82 |
| aug-pc-2 | | 1.3341 | 1.0889 | 121.79 | 1634.92 | 1591.22 | 1.3244 | 1.0824 | 121.74 | | 1689.47 | 1646.40 | 184 |
| aug-pc-3 | | 1.3337 | 1.0882 | 121.79 | 1634.71 | 1592.02 | 1.32411 | 1.0819 | 121.74 | | 1689.18 | 1665.43 | 378 |
| aug-pc-4 | | 1.3337 | 1.0882 | 121.79 | 1634.77 | 1592.36 | 1.3241 | 1.0819 | 121.74 | | 1689.13 | 1646.33 | 642 |
| **CBS(apcn)** | | **1.3334** | **1.0877** | **121.79** | **1634.62** | **1593.04** | **1.3239** | **1.0815** | **121.74** | | **1688.90** | **1644.54** |  |
| **Exp^a^.** | | **1.3305** | **1.0805** | **121.45** | **1656.40** | **1625.40** | **1.3305** | **1.0805** | **121.45** | | **1656.40** | **1625.40** |  |
|  | |  |  |  |  |  |  |  |  | |  |  |  |
| **Tab. S2A.** Continuation | | **BLYP** | | | | | **B3LYP** | | | | | |  |
| BASIS SET | | Structure | | | str C=C | | Structure | | | | str C=C | |  |
|  | | C=C [Å] | C-H [Å] | HCH | harm | anharm | C=C [Å] | C-H [Å] | | HCH | harm | anharm |  |
| **Deviation from Exp.** | | | | | | | | | | | | |  |
| **6-311++G**** | 0.0079 | | 0.0110 | 0.34 | 3.14 | -38.23 | -0.0016 | 0.0045 | | 0.29 | 58.14 | 15.87 |  |
| 6-311++G (3df,2pd) | 0.0036 | | 0.0082 | 0.34 | 10.06 | -33.70 | -0.0058 | 0.0018 | | 0.29 | 63.84 | 21.00 |  |
| pc-0 | 0.0218 | | 0.0197 | 0.08 | -0.41 | -45.78 | 0.0128 | 0.0121 | | 0.04 | 50.32 | 6.84 |  |
| pc-1 | 0.0093 | | 0.0179 | 0.31 | 16.53 | -27.62 | -0.0007 | 0.0104 | | 0.28 | 74.08 | 28.33 |  |
| pc-2 | 0.0035 | | 0.0084 | 0.35 | 10.59 | -33.06 | -0.0062 | 0.0019 | | 0.30 | 65.10 | 22.02 |  |
| pc-3 | 0.0032 | | 0.0077 | 0.34 | 9.37 | -33.75 | -0.0064 | 0.0014 | | 0.29 | 63.84 | 20.91 |  |
| pc-4 | 0.0032 | | 0.0077 | 0.34 | 9.38 | -33.13 | -0.0064 | 0.0014 | | 0.29 | 63.73 | 20.53 |  |
| **CBS(pcn)** | **0.0030** | | **0.0072** | **0.33** | **8.44** | **-33.52** | **-0.0065** | **0.0010** | | **0.28** | **62.76** | **20.59** |  |
| aug-pc-0 | 0.0251 | | 0.0199 | 0.01 | -13.33 | -56.32 | 0.0157 | 0.0124 | | 0.01 | 37.51 | -3.77 |  |
| aug-pc-1 | 0.0104 | | 0.0165 | 0.29 | 9.56 | -34.30 | 0.0003 | 0.0093 | | 0.25 | 66.63 | 21.33 |  |
| aug-pc-2 | 0.0036 | | 0.0084 | 0.34 | 9.52 | -34.18 | -0.0061 | 0.0019 | | 0.29 | 64.07 | 21.00 |  |
| aug-pc-3 | 0.0032 | | 0.0077 | 0.34 | 9.31 | -33.38 | -0.0064 | 0.0014 | | 0.29 | 63.78 | 40.03 |  |
| aug-pc-4 | 0.0032 | | 0.0077 | 0.34 | 9.37 | -33.04 | -0.0064 | 0.0014 | | 0.29 | 63.73 | 20.93 |  |
| **CBS(apcn)** | **0.0029** | | **0.0072** | **0.34** | **9.22** | **-32.36** | **-0.0066** | **0.0010** | | **0.29** | **63.50** | **19.14** |  |

a.) Semi-experimental structural parameters from ref. 43, 45, 46 and observed harmonic and fundamental frequency from ref. 62, 63, 72.

**Tab. S2B.** Structural parameters and harmonic and anharmonic stretch C=C mode of ethylene recalculated with B3LYP and Jensen’s segmented^ab^ basis sets. The number of basis functions (B. f.), cpu time for optimization and anharmonic frequency calculation^c^, as well as the CBS values for the largest Jensen basis sets (estimated from Eq. 2) are also given. The deviations from experimental values are also shown.

| Basis set | Structure[Å and deg] | | | ν(C=C) | | E | B. f. | cpu time | |
| --- | --- | --- | --- | --- | --- | --- | --- | --- | --- |
|  | C=C | C-H | HCH | harm | anharm |  |  | opt | anharm |
| pc-2 | 1.3246 | 1.0824 | 121.752 | 1689.8 | 1646.7 | -78.62587350 | 116 | 1m 4s | 4h 57m |
| pc-3 | 1.3241 | 1.0819 | 121.738 | 1689.3 | 1647.4 | -78.63148500 | 264 | 18m 18s | 2d 2h 4m |
| pc-4 | 1.3241 | 1.0818 | 121.735 | 1689.2 | 1645.8 | -78.63171929 | 470 | 1h 41m | 18d 5h 30m |
| **CBS(pcn)** | **1.3241** | **1.0818** | **121.734** | **1689.1** | **1644.7** | **-78.63189000** |  |  |  |
|  |  |  |  |  |  |  |  |  |  |
| aug-pc-2 | 1.3247 | 1.0824 | 121.739 | 1688.9 | 1645.8 | -78.62605250 | 184 | 3m 53s | 17h 41m |
| aug-pc-3 | 1.3241 | 1.0819 | 121.736 | 1689.2 | 1647.8 | -78.63150110 | 378 | 46m 46s | 5d 24m |
| aug-pc-4 | 1.3241 | 1.0818 | 121.735 | 1689.1 | 1645.9 | -78.63172550 | 642 | 1d 7h 28m | 79d 8h |
| **CBS(apcn)** | **1.3241** | **1.0818** | **121.735** | **1689.1** | **1644.6** | **-78.63189000** |  |  |  |
| ***Exp^d^.*** | ***1.3305*** | ***1.0805*** | ***121.45*** | ***1656.4*** | ***1625.4*** |  |  |  |  |
| **Deviation from Exp.** | | | | | |  |  |  |  |
| **CBS(pcn)** | -0.0064 | 0.0013 | 0.284 | 63.7 | 19.3 |  |  |  |  |
| **CBS(apcn)** | -0.0064 | 0.0013 | 0.285 | 63.7 | 19.2 |  |  |  |  |

a) downloaded as optimized general contracted; b) calculated using 4 processors and 4GB memory; c) abbreviations d, h, m and s stand for day, hour, minute and second, respectively; d) Semi experimental structural parameters from ref. 43, 45, 46, observed harmonic and fundamental frequency from 62, 63, 72. Calculations using 4 processors and 4 GB memory.

**Tab. S3A.** BLYP calculated harmonic vibrational frequencies of ethylene^a^ using polarization-consistent basis sets

| **-2** | **-1** | **pc-0** | **pc-1** | **pc-2** | **pc-3** | **pc-4** | **apc-0** | **apc-1** | **apc-2** | **apc-3** | **apc-4** |
| --- | --- | --- | --- | --- | --- | --- | --- | --- | --- | --- | --- |
| 3142.03 | 3146.59 | 3165.18 | 3150.75 | 3149.84 | 3143.94 | 3143.64 | 3167.38 | 3146.64 | 3148.67 | 3143.94 | 3143.64 |
| 3112.34 | 3117.99 | 3130.05 | 3124.23 | 3120.64 | 3115.27 | 3114.98 | 3133.96 | 3118.40 | 3119.78 | 3115.27 | 3114.98 |
| 3058.43 | 3063.62 | 3083.52 | 3064.82 | 3066.35 | 3061.64 | 3061.33 | 3075.56 | 3059.75 | 3065.43 | 3061.67 | 3061.33 |
| 3046.23 | 3051.89 | 3066.99 | 3050.73 | 3054.99 | 3050.97 | 3050.68 | 3060.81 | 3046.90 | 3054.25 | 3050.98 | 3050.68 |
| 1628.54 | 1635.46 | 1624.99 | 1641.93 | 1635.99 | 1634.77 | 1634.77 | 1612.07 | 1634.96 | 1634.92 | 1634.71 | 1634.77 |
| 1437.08 | 1445.67 | 1465.41 | 1421.89 | 1444.87 | 1446.49 | 1446.41 | 1461.03 | 1423.47 | 1444.74 | 1446.41 | 1446.41 |
| 1342.73 | 1347.08 | 1353.90 | 1339.28 | 1347.41 | 1346.70 | 1346.59 | 1340.56 | 1337.11 | 1346.35 | 1346.63 | 1346.59 |
| 1206.80 | 1216.17 | 1224.21 | 1202.13 | 1216.30 | 1217.67 | 1217.56 | 1224.56 | 1202.49 | 1215.91 | 1217.58 | 1217.56 |
| 1029.00 | 1034.52 | 1029.56 | 1028.60 | 1035.07 | 1035.82 | 1035.88 | 1016.97 | 1018.33 | 1034.77 | 1035.78 | 1035.88 |
| 940.52 | 946.07 | 956.69 | 934.18 | 946.78 | 945.94 | 945.85 | 962.95 | 939.49 | 945.62 | 945.89 | 945.85 |
| 932.33 | 942.03 | 947.61 | 916.44 | 943.69 | 943.88 | 943.77 | 947.35 | 926.79 | 942.92 | 943.56 | 943.77 |
| 816.00 | 818.94 | 837.22 | 816.49 | 818.54 | 818.88 | 818.74 | 843.48 | 813.98 | 817.79 | 818.80 | 818.74 |

**Tab. S3B**. BLYP calculated anharmonic vibrational frequencies of ethylene^a^ using polarization-consistent basis sets

| **-2** | **-1** | **pc-0** | **pc-1** | **pc-2** | **pc-3** | **pc-4** | **apc-0** | **apc-1** | **apc-2** | **apc-3** | **apc-4** |
| --- | --- | --- | --- | --- | --- | --- | --- | --- | --- | --- | --- |
| 2993.60 | 2997.47 | 3001.47 | 2997.82 | 2998.70 | 2995.16 | 2994.94 | 3003.84 | 2993.68 | 2997.76 | 2995.23 | 2994.95 |
| 2966.33 | 2970.99 | 2968.98 | 2972.90 | 2971.86 | 2968.90 | 2968.80 | 2972.55 | 2968.15 | 2971.19 | 2968.75 | 2968.86 |
| 2918.29 | 2923.76 | 2927.56 | 2921.16 | 2924.55 | 2922.17 | 2921.84 | 2918.67 | 2916.30 | 2923.78 | 2927.00 | 2921.87 |
| 2887.43 | 2893.91 | 2894.65 | 2888.67 | 2894.97 | 2893.39 | 2894.23 | 2888.35 | 2884.65 | 2894.16 | 2893.60 | 2894.28 |
| 1587.18 | 1592.42 | 1579.62 | 1597.78 | 1592.35 | 1591.65 | 1592.28 | 1569.08 | 1591.10 | 1591.22 | 1592.02 | 1592.36 |
| 1402.72 | 1409.06 | 1429.50 | 1388.44 | 1407.51 | 1409.63 | 1415.90 | 1426.70 | 1390.87 | 1407.26 | 1410.29 | 1416.03 |
| 1318.17 | 1321.31 | 1329.32 | 1313.66 | 1321.14 | 1320.83 | 1321.37 | 1317.42 | 1310.49 | 1320.02 | 1321.14 | 1321.43 |
| 1185.68 | 1191.76 | 1203.69 | 1179.65 | 1191.16 | 1192.96 | 1193.25 | 1203.65 | 1179.70 | 1190.80 | 1195.07 | 1193.51 |
| 1005.37 | 1011.14 | 1006.43 | 1005.26 | 1010.40 | 1011.64 | 1013.10 | 992.53 | 1002.26 | 1009.99 | 1012.85 | 1013.39 |
| 925.09 | 930.58 | 943.05 | 920.05 | 929.94 | 930.30 | 931.58 | 933.47 | 917.47 | 929.07 | 930.43 | 931.67 |
| 915.83 | 925.52 | 937.46 | 902.29 | 924.66 | 925.92 | 929.25 | 931.59 | 891.38 | 924.44 | 932.80 | 929.37 |
| 814.54 | 814.87 | 838.23 | 811.42 | 813.51 | 814.76 | 815.05 | 842.15 | 810.36 | 812.93 | 814.86 | 815.11 |

1. Table headings –2 and –1 correspond to 6-311++G** and 6-311++G(3df,2pd) basis sets.

**Tab. S4A**. BLYP calculated harmonic vibrational frequencies of ethylene using Dunning’s correlation-consistent basis sets

| **DZ** | **TZ** | **QZ** | **5Z** | **6Z** | **aDZ** | **aTZ** | **aQZ** | **a5Z** | **a6Z** |
| --- | --- | --- | --- | --- | --- | --- | --- | --- | --- |
| 3150.67 | 3144.82 | 3142.50 | 3143.69 | 3143.56 | 3159.06 | 3143.16 | 3143.05 | 3143.58 | 3143.59 |
| 3121.91 | 3115.47 | 3113.63 | 3115.05 | 3114.91 | 3130.45 | 3114.02 | 3114.35 | 3114.87 | 3114.92 |
| 3059.58 | 3063.34 | 3060.93 | 3061.42 | 3061.27 | 3067.71 | 3062.99 | 3061.32 | 3061.34 | 3061.29 |
| 3044.70 | 3051.83 | 3050.08 | 3050.72 | 3050.61 | 3055.76 | 3051.31 | 3050.48 | 3050.63 | 3050.62 |
| 1638.56 | 1639.01 | 1635.98 | 1635.29 | 1634.80 | 1627.36 | 1634.89 | 1634.35 | 1634.86 | 1634.72 |
| 1407.43 | 1445.59 | 1445.45 | 1446.59 | 1446.42 | 1423.03 | 1445.39 | 1446.15 | 1446.38 | 1446.44 |
| 1330.12 | 1348.27 | 1346.77 | 1346.85 | 1346.63 | 1333.77 | 1345.95 | 1346.64 | 1346.60 | 1346.59 |
| 1199.16 | 1216.48 | 1215.44 | 1217.58 | 1217.53 | 1197.70 | 1214.46 | 1217.52 | 1217.31 | 1217.55 |
| 1029.14 | 1038.86 | 1036.64 | 1036.13 | 1035.85 | 1015.88 | 1032.43 | 1035.24 | 1035.68 | 1035.93 |
| 935.88 | 945.33 | 946.10 | 945.89 | 945.86 | 951.93 | 945.90 | 946.28 | 945.62 | 945.89 |
| 916.87 | 939.10 | 943.05 | 943.47 | 943.72 | 946.15 | 941.57 | 943.97 | 943.23 | 943.88 |
| 806.54 | 818.20 | 817.22 | 818.83 | 818.77 | 806.62 | 815.72 | 818.07 | 818.59 | 818.72 |

**Tab. S4B.** BLYP calculated anharmonic vibrational frequencies of ethylene using Dunning’s correlation-consistent basis sets

| **DZ** | **TZ** | **QZ** | **5Z** | **6Z** | **aDZ** | **aTZ** | **aQZ** | **a5Z** | **a6Z** |
| --- | --- | --- | --- | --- | --- | --- | --- | --- | --- |
| 2993.15 | 2996.16 | 2993.83 | 2994.82 | 2996.45 | 3002.54 | 2995.20 | 2993.89 | 2995.05 | 2992.13 |
| 2966.87 | 2969.60 | 2967.95 | 2968.60 | 2969.78 | 2975.45 | 2969.23 | 2967.30 | 2969.21 | 2965.88 |
| 2913.41 | 2924.43 | 2921.74 | 2921.88 | 2922.92 | 2920.00 | 2922.72 | 2921.21 | 2921.93 | 2916.40 |
| 2878.70 | 2894.94 | 2892.85 | 2893.11 | 2893.97 | 2886.83 | 2893.64 | 2892.55 | 2892.65 | 2886.92 |
| 1589.44 | 1595.28 | 1593.11 | 1592.02 | 1590.39 | 1583.34 | 1591.41 | 1591.83 | 1591.34 | 1588.09 |
| 1374.27 | 1408.56 | 1409.22 | 1409.46 | 1408.45 | 1387.48 | 1407.93 | 1410.19 | 1407.81 | 1407.48 |
| 1303.12 | 1321.99 | 1321.25 | 1320.92 | 1320.16 | 1308.42 | 1319.97 | 1321.27 | 1321.14 | 1318.57 |
| 1173.51 | 1191.73 | 1194.47 | 1192.76 | 1195.22 | 1176.26 | 1195.28 | 1193.54 | 1194.09 | 1189.43 |
| 1001.95 | 1014.42 | 1013.53 | 1011.61 | 1012.12 | 993.88 | 1020.83 | 1011.99 | 1007.97 | 1006.54 |
| 915.56 | 929.19 | 930.43 | 930.17 | 931.72 | 926.59 | 929.92 | 929.76 | 930.72 | 923.53 |
| 894.14 | 921.74 | 925.46 | 926.23 | 925.97 | 914.77 | 927.01 | 925.48 | 928.11 | 917.27 |
| 799.10 | 813.47 | 814.80 | 814.58 | 809.90 | 804.60 | 815.38 | 814.36 | 810.96 | 806.65 |

**Tab. S5A.** B3LYP calculated harmonic vibrational frequencies of ethylene^a^ using polarization-consistent basis sets

| **-2** | **-1** | **pc-0** | **pc-1** | **pc-2** | **pc-3** | **pc-4** | **apc-0** | **apc-1** | **apc-2** | **apc-3** | **apc-4** |
| --- | --- | --- | --- | --- | --- | --- | --- | --- | --- | --- | --- |
| 3222.03 | 3224.54 | 3254.40 | 3237.70 | 3229.45 | 3223.11 | 3222.89 | 3254.76 | 3230.97 | 3228.48 | 3223.10 | 3222.89 |
| 3193.62 | 3196.84 | 3220.17 | 3212.28 | 3201.21 | 3195.32 | 3195.11 | 3222.41 | 3203.16 | 3200.52 | 3195.32 | 3195.11 |
| 3136.28 | 3139.71 | 3170.80 | 3149.74 | 3144.12 | 3138.85 | 3138.61 | 3161.00 | 3142.40 | 3143.21 | 3138.86 | 3138.61 |
| 3121.86 | 3125.93 | 3152.37 | 3133.80 | 3130.62 | 3126.02 | 3125.79 | 3144.57 | 3126.99 | 3129.80 | 3126.02 | 3125.79 |
| 1683.54 | 1689.24 | 1675.72 | 1699.48 | 1690.50 | 1689.24 | 1689.13 | 1662.91 | 1692.03 | 1689.47 | 1689.18 | 1689.13 |
| 1471.51 | 1479.36 | 1500.98 | 1460.15 | 1479.44 | 1480.49 | 1480.42 | 1496.36 | 1460.48 | 1479.11 | 1480.43 | 1480.42 |
| 1377.31 | 1381.25 | 1389.81 | 1376.12 | 1382.10 | 1381.28 | 1381.17 | 1376.86 | 1373.42 | 1381.20 | 1381.24 | 1381.17 |
| 1238.06 | 1246.52 | 1258.36 | 1235.14 | 1247.10 | 1248.12 | 1248.04 | 1258.32 | 1235.11 | 1246.71 | 1248.07 | 1248.04 |
| 1058.47 | 1063.50 | 1059.64 | 1058.18 | 1064.48 | 1064.86 | 1064.93 | 1047.89 | 1042.84 | 1063.92 | 1064.86 | 1064.92 |
| 976.58 | 985.50 | 1012.04 | 969.71 | 988.32 | 987.50 | 987.46 | 1018.53 | 971.91 | 986.52 | 987.22 | 987.46 |
| 974.54 | 979.54 | 988.21 | 963.38 | 980.97 | 979.68 | 979.60 | 988.39 | 966.99 | 979.73 | 979.66 | 979.60 |
| 834.63 | 836.75 | 862.33 | 836.65 | 836.89 | 836.96 | 836.85 | 867.90 | 833.03 | 836.20 | 836.90 | 836.85 |

**Tab. S5B.** B3LYP calculated anharmonic vibrational frequencies of ethylene^a^ using polarization-consistent basis sets

| **-2** | **-1** | **pc-0** | **pc-1** | **pc-2** | **pc-3** | **pc-4** | **apc-0** | **apc-1** | **apc-2** | **apc-3** | **apc-4** |
| --- | --- | --- | --- | --- | --- | --- | --- | --- | --- | --- | --- |
| 3078.17 | 3081.22 | 3093.89 | 3089.32 | 3083.77 | 3078.69 | 3078.15 | 3094.59 | 3083.13 | 3082.80 | 3088.54 | 3078.37 |
| 3052.45 | 3056.03 | 3062.40 | 3065.84 | 3058.26 | 3053.83 | 3053.92 | 3064.43 | 3059.03 | 3057.50 | 3063.31 | 3054.26 |
| 3002.37 | 3006.91 | 3018.51 | 3011.74 | 3009.07 | 3005.06 | 3005.08 | 3008.07 | 3005.21 | 3008.22 | 3014.80 | 3005.21 |
| 2969.75 | 2975.54 | 2983.31 | 2978.13 | 2977.98 | 2974.35 | 2974.19 | 2975.84 | 2972.18 | 2977.14 | 2987.89 | 2974.45 |
| 1641.27 | 1646.81 | 1632.24 | 1653.73 | 1647.42 | 1646.31 | 1645.93 | 1621.64 | 1646.74 | 1646.41 | 1665.43 | 1646.33 |
| 1437.94 | 1444.94 | 1466.59 | 1428.12 | 1444.33 | 1444.52 | 1443.78 | 1463.98 | 1429.62 | 1444.05 | 1479.70 | 1444.29 |
| 1353.33 | 1356.81 | 1366.02 | 1351.30 | 1357.30 | 1355.46 | 1355.84 | 1354.58 | 1347.99 | 1356.34 | 1378.62 | 1356.06 |
| 1216.80 | 1223.84 | 1238.47 | 1213.99 | 1223.70 | 1223.32 | 1223.62 | 1238.16 | 1213.55 | 1223.53 | 1257.71 | 1222.47 |
| 1035.32 | 1040.60 | 1037.10 | 1034.57 | 1040.38 | 1041.27 | 1036.37 | 1023.77 | 1033.21 | 1040.36 | 1075.99 | 1037.60 |
| 960.02 | 968.80 | 997.12 | 954.84 | 968.96 | 972.23 | 964.58 | 986.14 | 951.07 | 968.97 | 1245.51 | 965.52 |
| 959.37 | 963.99 | 977.62 | 948.50 | 964.35 | 964.72 | 961.33 | 971.63 | 937.64 | 963.37 | 1000.89 | 961.90 |
| 833.11 | 835.43 | 863.02 | 833.54 | 834.69 | 836.30 | 834.35 | 866.26 | 830.91 | 834.21 | 874.79 | 835.11 |

1. Table headings –2 and –1 correspond to 6-311++G** and 6-311++G(3df,2pd) basis sets.

**Tab. S6A**. B3LYP calculated harmonic vibrational frequencies of ethylene using segmented polarization-consistent basis sets

| **pcseg-0** | **pcseg-1** | **pcseg-2** | **pcseg-3** | **pcseg-4** | **apcseg-0** | **apcseg-1** | **apcseg-2** | **apcseg-3** | **apcseg-4** |
| --- | --- | --- | --- | --- | --- | --- | --- | --- | --- |
| 3234.97 | 3238.56 | 3228.81 | 3223.85 | 3223.28 | 3240.68 | 3230.88 | 3228.34 | 3223.74 | 3223.20 |
| 3205.03 | 3213.13 | 3200.42 | 3196.01 | 3195.43 | 3210.23 | 3203.02 | 3200.23 | 3195.93 | 3195.33 |
| 3155.47 | 3150.16 | 3143.85 | 3139.37 | 3139.03 | 3158.09 | 3141.98 | 3143.15 | 3139.32 | 3138.97 |
| 3145.04 | 3134.17 | 3130.64 | 3126.55 | 3126.14 | 3148.41 | 3126.55 | 3129.96 | 3126.48 | 3126.07 |
| 1704.90 | 1698.79 | 1689.81 | 1689.29 | 1689.16 | 1685.13 | 1690.88 | 1688.92 | 1689.23 | 1689.14 |
| 1520.46 | 1459.69 | 1479.28 | 1480.21 | 1480.51 | 1512.15 | 1459.81 | 1478.75 | 1480.20 | 1480.52 |
| 1410.44 | 1374.71 | 1381.59 | 1381.25 | 1381.25 | 1401.76 | 1372.42 | 1380.77 | 1381.21 | 1381.23 |
| 1277.65 | 1234.41 | 1246.78 | 1247.93 | 1248.05 | 1275.44 | 1233.89 | 1246.30 | 1247.93 | 1248.04 |
| 1077.28 | 1057.80 | 1064.38 | 1064.88 | 1064.94 | 1061.39 | 1041.12 | 1063.63 | 1064.85 | 1064.87 |
| 1035.34 | 969.17 | 988.49 | 987.60 | 987.43 | 1050.91 | 971.99 | 987.24 | 987.42 | 987.35 |
| 1008.38 | 962.45 | 980.49 | 979.74 | 979.63 | 1015.95 | 966.04 | 979.41 | 979.73 | 979.66 |
| 875.27 | 835.70 | 836.42 | 836.84 | 836.92 | 879.62 | 831.20 | 835.85 | 836.84 | 836.91 |

**Tab. S6B.** B3LYP calculated anharmonic vibrational frequencies of ethylene using segmented polarization-consistent basis sets

| **pcseg-0** | **pcseg-1** | **pcseg-2** | **pcseg-3** | **pcseg-4** | **apcseg-0** | **apcseg-1** | **apcseg-2** | **apcseg-3** | **apcseg-4** |
| --- | --- | --- | --- | --- | --- | --- | --- | --- | --- |
| 3067.81 | 3089.32 | 3082.88 | 3080.96 | 3077.49 | 3073.56 | 3082.88 | 3082.22 | 3081.08 | 3075.83 |
| 3041.23 | 3065.81 | 3057.34 | 3056.10 | 3051.94 | 3045.63 | 3059.14 | 3056.93 | 3055.64 | 3050.32 |
| 3001.44 | 3011.48 | 3008.65 | 3007.68 | 3004.05 | 3001.07 | 3004.38 | 3007.84 | 3007.26 | 2998.19 |
| 2976.34 | 2977.78 | 2977.66 | 2977.82 | 2973.86 | 2977.17 | 2971.46 | 2976.79 | 2978.29 | 2973.04 |
| 1660.75 | 1652.70 | 1646.75 | 1647.39 | 1645.84 | 1642.68 | 1645.42 | 1645.85 | 1647.75 | 1645.47 |
| 1484.34 | 1427.57 | 1444.09 | 1444.86 | 1443.54 | 1478.44 | 1429.29 | 1443.70 | 1441.74 | 1445.57 |
| 1385.04 | 1350.00 | 1356.76 | 1356.27 | 1355.05 | 1376.91 | 1346.81 | 1355.97 | 1356.60 | 1355.69 |
| 1253.48 | 1213.31 | 1223.27 | 1223.84 | 1221.13 | 1250.32 | 1213.23 | 1222.91 | 1224.46 | 1222.39 |
| 1053.71 | 1034.14 | 1040.22 | 1041.24 | 1040.05 | 1036.04 | 1034.65 | 1040.45 | 1045.63 | 1039.85 |
| 1017.98 | 954.24 | 969.13 | 971.52 | 972.19 | 1006.83 | 950.84 | 968.81 | 979.94 | 981.30 |
| 994.54 | 947.56 | 963.88 | 964.42 | 963.73 | 993.84 | 938.29 | 963.10 | 965.52 | 960.83 |
| 871.60 | 832.71 | 834.28 | 836.18 | 836.90 | 873.69 | 830.52 | 833.85 | 836.78 | 833.15 |

**Tab. S7A.** B3LYP calculated harmonic vibrational frequencies of ethylene using Dunning’s correlation-consistent basis sets

| **DZ** | **TZ** | **QZ** | **5Z** | **6Z** | **aDZ** | **aTZ** | **aQZ** | **a5Z** | **a6Z** |
| --- | --- | --- | --- | --- | --- | --- | --- | --- | --- |
| 3235.67 | 3223.22 | 3221.61 | 3222.87 | 3222.81 | 3240.65 | 3222.69 | 3222.05 | 3222.78 | 3222.81 |
| 3208.12 | 3194.77 | 3193.64 | 3195.11 | 3195.04 | 3212.71 | 3194.41 | 3194.18 | 3194.97 | 3195.03 |
| 3143.11 | 3139.40 | 3137.85 | 3138.62 | 3138.53 | 3147.70 | 3139.60 | 3138.11 | 3138.54 | 3138.53 |
| 3126.08 | 3125.63 | 3124.86 | 3125.76 | 3125.71 | 3133.43 | 3125.90 | 3125.14 | 3125.68 | 3125.70 |
| 1695.74 | 1692.78 | 1690.13 | 1689.47 | 1689.14 | 1683.68 | 1688.85 | 1688.57 | 1689.15 | 1689.09 |
| 1444.59 | 1478.88 | 1479.49 | 1480.49 | 1480.41 | 1458.32 | 1479.21 | 1480.07 | 1480.35 | 1480.42 |
| 1365.79 | 1382.12 | 1381.17 | 1381.31 | 1381.18 | 1369.23 | 1380.52 | 1381.08 | 1381.14 | 1381.16 |
| 1229.15 | 1246.56 | 1246.24 | 1248.02 | 1248.02 | 1229.02 | 1245.51 | 1247.90 | 1247.86 | 1248.04 |
| 1057.44 | 1067.23 | 1065.56 | 1065.08 | 1064.92 | 1045.41 | 1060.45 | 1064.26 | 1064.73 | 1064.95 |
| 968.25 | 983.69 | 986.93 | 987.29 | 987.45 | 985.61 | 984.98 | 987.47 | 987.00 | 987.53 |
| 959.46 | 979.14 | 979.76 | 979.64 | 979.62 | 983.01 | 979.15 | 979.92 | 979.41 | 979.63 |
| 825.89 | 836.09 | 835.69 | 836.90 | 836.88 | 825.80 | 834.39 | 836.33 | 836.71 | 836.82 |

**Tab. S7B.** B3LYP calculated anharmonic vibrational frequencies of ethylene using Dunning’s correlation-consistent basis sets

| **DZ** | **TZ** | **QZ** | **5Z** | **6Z** | **aDZ** | **aTZ** | **aQZ** | **a5Z** | **a6Z** |
| --- | --- | --- | --- | --- | --- | --- | --- | --- | --- |
| 3082.92 | 3080.25 | 3077.46 | 3078.36 | 3083.56 | 3088.65 | 3080.77 | 3077.46 | 3078.07 | 3075.117 |
| 3058.15 | 3055.03 | 3053.01 | 3053.52 | 3058.87 | 3062.72 | 3056.80 | 3052.85 | 3052.01 | 3051.936 |
| 3002.52 | 3007.62 | 3004.54 | 3004.93 | 3009.63 | 3005.80 | 3006.64 | 3004.10 | 3004.88 | 3001.187 |
| 2967.12 | 2976.50 | 2973.55 | 2974.25 | 2978.08 | 2971.64 | 2976.04 | 2972.96 | 2975.64 | 2971.285 |
| 1662.81 | 1649.50 | 1645.67 | 1646.35 | 1648.67 | 1637.20 | 1647.27 | 1644.03 | 1649.41 | 1642.194 |
| 1412.36 | 1444.54 | 1443.12 | 1444.51 | 1446.52 | 1423.93 | 1444.62 | 1442.30 | 1448.59 | 1439.091 |
| 1339.47 | 1357.61 | 1354.42 | 1355.06 | 1357.86 | 1344.37 | 1356.56 | 1353.58 | 1356.42 | 1354.234 |
| 1204.92 | 1223.76 | 1223.61 | 1223.99 | 1227.49 | 1208.00 | 1225.95 | 1220.26 | 1227.09 | 1213.062 |
| 1030.83 | 1043.54 | 1039.93 | 1042.62 | 1046.73 | 1022.90 | 1063.14 | 1033.70 | 1044.33 | 1034.217 |
| 948.23 | 965.80 | 968.10 | 967.78 | 972.82 | 955.96 | 970.80 | 970.04 | 978.83 | 948.183 |
| 937.63 | 963.15 | 963.60 | 964.10 | 965.81 | 958.68 | 963.97 | 963.70 | 969.69 | 960.046 |
| 819.92 | 834.28 | 834.37 | 836.24 | 837.80 | 824.56 | 837.15 | 835.36 | 847.99 | 829.875 |

**Tab. S8A.** CBS estimated individual ethylene harmonic and anharmonic vibrational frequencies calculated using two

density functionals and selected basis sets^a^

| **EXP.** | **BLYP/Polarized** | | | | **BLYP/Dunning** | | | | **B3LYP/segmented** | | | |
| --- | --- | --- | --- | --- | --- | --- | --- | --- | --- | --- | --- | --- |
|  | Harmonic | | Anharmonic | | Harmonic | | Anharmonic | | Harmonic | | Anharmonic | |
|  | pcn | apcn | pcn | apcn | XZ | aXZ | XZ | aXZ | pcsegn | apcsegn | pcsegn | apcsegn |
| 3104.89 | 3143.41 | 3143.41 | 2994.77 | 2994.75 | 3143.39 | 3143.60 | 2998.69 | 2992.68 | 3222.49 | 3222.45 | 3072.72 | 3068.62 |
| 3083.36 | 3114.77 | 3114.76 | 2968.72 | 2968.95 | 3114.72 | 3114.99 | 2971.40 | 2966.86 | 3194.62 | 3194.52 | 3046.23 | 3043.02 |
| 3022.03 | 3061.10 | 3061.08 | 2921.60 | 2918.13 | 3061.05 | 3061.22 | 2924.36 | 2916.58 | 3138.56 | 3138.49 | 2999.06 | 2985.74 |
| 2988.64 | 3050.47 | 3050.46 | 2894.84 | 2894.78 | 3050.47 | 3050.61 | 2895.16 | 2886.73 | 3125.56 | 3125.51 | 2968.43 | 2965.82 |
| 1625.4 | 1634.76 | 1634.81 | 1592.73 | 1592.60 | 1634.14 | 1634.54 | 1588.14 | 1587.65 | 1688.98 | 1689.03 | 1643.71 | 1642.34 |
| 1442.47 | 1446.35 | 1446.40 | 1420.47 | 1420.22 | 1446.18 | 1446.51 | 1407.05 | 1406.07 | 1480.92 | 1480.96 | 1441.73 | 1450.84 |
| 1343.54 | 1346.52 | 1346.57 | 1321.76 | 1321.64 | 1346.33 | 1346.58 | 1319.12 | 1318.37 | 1381.25 | 1381.26 | 1353.37 | 1354.45 |
| 1222 | 1217.48 | 1217.55 | 1193.46 | 1192.37 | 1217.46 | 1217.88 | 1198.59 | 1189.55 | 1248.22 | 1248.18 | 1217.41 | 1219.54 |
| 1025.59 | 1035.92 | 1035.95 | 1014.17 | 1013.78 | 1035.46 | 1036.29 | 1012.81 | 1004.12 | 1065.03 | 1064.90 | 1038.41 | 1031.91 |
| 948.77 | 945.78 | 945.81 | 932.52 | 932.57 | 945.82 | 946.27 | 933.85 | 923.33 | 987.19 | 987.26 | 973.12 | 983.15 |
| 939.86 | 943.69 | 943.92 | 931.69 | 926.86 | 944.07 | 944.77 | 925.61 | 918.33 | 979.49 | 979.56 | 962.77 | 954.39 |
| 825.93 | 818.64 | 818.70 | 815.26 | 815.29 | 818.69 | 818.90 | 803.47 | 804.46 | 837.04 | 837.01 | 837.88 | 828.16 |
|  | **B3LYP/Polarized** | | | | B3LYP/Dunning | | | |  |  |  |  |
| 3104.89 | 3222.73 | 3222.73 | 3077.75 | 3070.94 | 3222.81 | 3222.81 | 3083.56 | 3072.02 |  |  |  |  |
| 3083.36 | 3194.96 | 3194.96 | 3053.98 | 3047.65 | 3195.04 | 3195.03 | 3058.87 | 3051.87 |  |  |  |  |
| 3022.03 | 3138.43 | 3138.43 | 3005.10 | 2998.21 | 3138.53 | 3138.53 | 3009.63 | 2997.31 |  |  |  |  |
| 2988.64 | 3125.62 | 3125.63 | 2974.08 | 2964.64 | 3125.71 | 3125.70 | 2978.08 | 2966.72 |  |  |  |  |
| 1625.4 | 1689.05 | 1689.10 | 1645.65 | **1632.40** | 1689.14 | 1689.09 | 1648.67 | 1634.62 |  |  |  |  |
| 1442.47 | 1480.36 | 1480.40 | 1443.24 | **1418.44** | 1480.41 | 1480.42 | 1446.52 | 1429.12 |  |  |  |  |
| 1343.54 | 1381.08 | 1381.11 | 1356.12 | **1339.60** | 1381.18 | 1381.16 | 1357.86 | 1351.94 |  |  |  |  |
| 1222 | 1247.98 | 1248.01 | 1223.84 | **1196.76** | 1248.02 | 1248.04 | 1227.49 | 1198.34 |  |  |  |  |
| 1025.59 | 1064.98 | 1064.97 | 1032.79 | **1009.58** | 1064.92 | 1064.95 | 1046.73 | 1023.61 |  |  |  |  |
| 948.77 | 987.42 | 987.63 | 959.00 | **761.20** | 987.45 | 987.53 | 972.82 | **916.03** |  |  |  |  |
| 939.86 | 979.55 | 979.56 | 958.86 | **933.45** | 979.62 | 979.63 | 965.81 | 949.94 |  |  |  |  |
| 825.93 | 836.78 | 836.82 | 832.93 | **806.16** | 836.88 | 836.82 | 837.80 | **810.87** |  |  |  |  |

1. Some anharmonic frequencies are unreliable due to bad performance of B3LYP/aug-pc-3 calculations (marked in red and bold)

**Tab. S8B.** RMS deviations between CBS estimated individual ethylene harmonic and anharmonic vibrational frequencies calculated using two density functionals and selected basis sets^a^ and experimental value

| **EXP.** | **BLYP/Polarized** | | | | **BLYP/Dunning** | | | | **B3LYP/segmented** | | | |
| --- | --- | --- | --- | --- | --- | --- | --- | --- | --- | --- | --- | --- |
|  | Harmonic | | Anharmonic | | Harmonic | | Anharmonic | | Harmonic | | Anharmonic | |
|  | pc-n | apc-n | pc-n | apc-n | XZ | aXZ | XZ | aXZ | pcseg-n | apcseg-n | pcseg-n | apcseg-n |
| 3104.89 | 38.52 | 38.52 | -110.12 | -110.14 | 38.50 | 38.71 | -106.20 | -112.21 | 117.60 | 117.56 | -32.17 | -36.27 |
| 3083.36 | 31.41 | 31.40 | -114.64 | -114.41 | 31.36 | 31.63 | -111.96 | -116.50 | 111.26 | 111.16 | -37.13 | -40.34 |
| 3022.03 | 39.07 | 39.05 | -100.43 | -103.90 | 39.02 | 39.19 | -97.67 | -105.45 | 116.53 | 116.46 | -22.97 | -36.29 |
| 2988.64 | 61.83 | 61.82 | -93.80 | -93.86 | 61.83 | 61.97 | -93.48 | -101.91 | 136.92 | 136.87 | -20.21 | -22.82 |
| 1625.4 | 9.36 | 9.41 | -32.67 | -32.80 | 8.74 | 9.14 | -37.26 | -37.75 | 63.58 | 63.63 | 18.31 | 16.94 |
| 1442.47 | 3.88 | 3.93 | -22.00 | -22.25 | 3.71 | 4.04 | -35.42 | -36.40 | 38.45 | 38.49 | -0.74 | 8.37 |
| 1343.54 | 2.98 | 3.03 | -21.78 | -21.90 | 2.79 | 3.04 | -24.42 | -25.17 | 37.71 | 37.72 | 9.83 | 10.91 |
| 1222 | -4.52 | -4.45 | -28.54 | -29.63 | -4.54 | -4.12 | -23.41 | -32.45 | 26.22 | 26.18 | -4.59 | -2.46 |
| 1025.59 | 10.33 | 10.36 | -11.42 | -11.81 | 9.87 | 10.70 | -12.78 | -21.47 | 39.44 | 39.31 | 12.82 | 6.32 |
| 948.77 | -2.99 | -2.96 | -16.25 | -16.20 | -2.95 | -2.50 | -14.92 | -24.99 | 38.42 | 38.49 | 24.35 | 34.38 |
| 939.86 | 3.83 | 4.06 | -8.17 | -13.00 | 4.21 | 4.91 | -14.25 | -21.53 | 39.63 | 39.70 | 22.91 | 14.53 |
| 825.93 | -7.29 | -7.23 | -10.67 | -10.64 | -7.24 | -7.03 | -22.46 | -21.47 | 11.11 | 11.08 | 11.95 | 2.23 |
| **RMS** | **26.04** | **26.04** | **62.96** | **63.53** | **25.99** | **26.13** | **62.56** | **67.15** | **76.86** | **76.83** | **20.87** | **23.63** |
|  | **B3LYP/Polarized** | | | | B3LYP/Dunning | | | |  |  |  |  |
| 3104.89 | 117.84 | 117.84 | -27.14 | -33.95 | 117.92 | 117.92 | -21.33 | -32.874 |  |  |  |  |
| 3083.36 | 111.60 | 111.60 | -29.38 | -35.71 | 111.68 | 111.67 | -24.49 | -31.497 |  |  |  |  |
| 3022.03 | 116.40 | 116.40 | -16.93 | -23.82 | 116.50 | 116.50 | -12.40 | -24.716 |  |  |  |  |
| 2988.64 | 136.98 | 136.99 | -14.56 | -24.00 | 137.07 | 137.06 | -10.56 | -21.924 |  |  |  |  |
| 1625.4 | 63.65 | 63.70 | 20.25 | 7.00 | 63.74 | 63.69 | 23.27 | 9.221 |  |  |  |  |
| 1442.47 | 37.89 | 37.93 | 0.77 | -24.03 | 37.94 | 37.95 | 4.05 | -13.347 |  |  |  |  |
| 1343.54 | 37.54 | 37.57 | 12.58 | -3.94 | 37.64 | 37.62 | 14.32 | 8.398 |  |  |  |  |
| 1222 | 25.98 | 26.01 | 1.84 | -25.24 | 26.02 | 26.04 | 5.49 | -23.659 |  |  |  |  |
| 1025.59 | 39.39 | 39.38 | 7.20 | -16.01 | 39.33 | 39.36 | 21.14 | -1.981 |  |  |  |  |
| 948.77 | 38.65 | 38.86 | 10.23 | -187.57 | 38.68 | 38.76 | 24.05 | **-32.744^a^** |  |  |  |  |
| 939.86 | 39.69 | 39.70 | 19.00 | -6.41 | 39.76 | 39.77 | 25.95 | 10.069 |  |  |  |  |
| 825.93 | 10.85 | 10.89 | 7.00 | -19.77 | 10.95 | 10.89 | 11.87 | **-15.057**^a^ |  |  |  |  |
| **RMS** | **76.90** | **76.92** | **16.42** | **58.25^a^** | **76.90** | **76.96** | **18.15** | **21.33^a^** |  |  |  |  |

1. Some anharmonic frequencies are unreliable due to bad performance of B3LYP/aug-pc-3 calculations and B3LYP/aug-cc-pVXZ (in bold)

**Tab. S9A.** CBS estimated ethylene raw and anharmonic ZPV energy calculated using two density functionals and selected basis sets^a^

| **N (X)** | **BLYP/Polarized** | | | | **BLYP/Dunning** | | | | **B3LYP/segmented** | | | |
| --- | --- | --- | --- | --- | --- | --- | --- | --- | --- | --- | --- | --- |
|  | Harmonic | | Anharmonic | | Harmonic | | Anharmonic | | Harmonic | | Anharmonic | |
|  | pcn | apcn | pcn | apcn | XZ | aXZ | XZ | aXZ | pcsegn | apcsegn | pcsegn | apcsegn |
| 0 (D) | 31.557 | 31.502 | 31.111 | 31.037 | 30.937 | 31.044 | 30.483 | 30.593 | 32.380 | 32.366 | 31.914 | 31.871 |
| 1 (T) | 31.278 | 31.246 | 30.844 | 30.800 | 31.116 | 31.090 | 30.686 | 30.679 | 31.922 | 31.836 | 31.499 | 31.422 |
| 2 (Q) | 31.408 | 31.393 | 30.964 | 30.952 | 31.099 | 31.104 | 30.674 | 30.672 | 31.981 | 31.969 | 31.558 | 31.546 |
| 3 (5) | 31.381 | 31.379 | 30.945 | 30.957 | 31.109 | 31.107 | 30.676 | 30.672 | 31.955 | 31.955 | 31.544 | 31.554 |
| 4 (6) | 31.377 | 31.377 | 30.952 | 30.955 | 31.107 | 31.108 | 30.676 | 30.636 | 31.953 | 31.953 | 31.527 | 31.525 |
| **CBS** | **31.361** | **31.368** | **30.941** | **30.957** | **31.112** | **31.109** | **30.678** | **30.587** | **31.937** | **31.944** | **31.518** | **31.528** |
|  | **B3LYP/Polarized** | | | | B3LYP/Dunning | | | |  |  |  |  |
| 0 (D) | 32.510 | 32.445 | 32.066 | 31.984 | 31.821 | 31.900 | 31.379 | 31.460 |  |  |  |  |
| 1 (T) | 32.203 | 32.126 | 31.779 | 31.704 | 31.950 | 31.931 | 31.537 | 31.549 |  |  |  |  |
| 2 (Q) | 32.264 | 32.249 | 31.837 | 31.825 | 31.941 | 31.943 | 31.518 | 31.511 |  |  |  |  |
| 3 (5) | 32.230 | 32.230 | 31.808 | **32.189** | 31.953 | 31.948 | 31.530 | 31.556 |  |  |  |  |
| 4 (6) | 32.228 | 32.228 | 31.794 | 31.796 | 31.950 | 31.950 | 31.558 | **31.479** |  |  |  |  |
| **CBS** | **32.208** | **32.216** | **31.776** | **~31.796** | **31.957** | **31.952** | **31.567** | **~ 31.5^a^** |  |  |  |  |

1. Some anharmonic ZPV values are unreliable due to bad performance of B3LYP/aug-pc-3 and B3LYP/cc-pVXZ,

as well as B3LYP/aug-cc-pVXZ calculations (marked in bold)
